# Delay in Diagnosis of Testicular Cancer; A Need for Awareness Programs

**DOI:** 10.1371/journal.pone.0141244

**Published:** 2015-11-25

**Authors:** Çiğdem Öztürk, Joke Fleer, Harald J. Hoekstra, Josette E. H. M. Hoekstra-Weebers

**Affiliations:** 1 Department of Surgical Oncology, University of Groningen, University Medical Center, Groningen, the Netherlands; 2 Department of Health Psychology, University of Groningen, University Medical Center, Groningen, the Netherlands; 3 Wenckebach Institute, University of Groningen, University Medical Center, Groningen, the Netherlands; University of Algarve, PORTUGAL

## Abstract

**Background Aim:**

To gain insight into patient and doctor delay in testicular cancer (TC) and factors associated with delay.

**Materials and Methods:**

Sixty of the 66 eligible men; median age 26 (range 17–45) years, diagnosed with TC at the University Medical Center Groningen completed a questionnaire on patients’ delay: interval from symptom onset to first consultation with a general practitioner (GP) and doctors’ delay: interval between GP and specialist visit.

**Results:**

Median patient reported delay was 30 (range 1–365) days. Patient delay and TC tumor stage were associated (p = .01). Lower educated men and men embarrassed about their scrotal change reported longer patient delay (r = -.25, r = .79 respectively). Age, marital status, TC awareness, warning signals, nor perceived limitations were associated with patient delay. Median patient reported time from GP to specialist (doctors’ delay) was 7 (range 0–240) days. Referral time and disease stage were associated (p = .04). Six patients never reported a scrotal change. Of the 54 patients reporting a testicular change, 29 (54%) patients were initially ‘misdiagnosed’, leading to a median doctors’ delay of 14 (1–240) days, which was longer (p< .001) than in the 25 (46%) patients whose GP suspected TC (median doctors’ delay 1(0–7 days).

**Conclusions:**

High variation in patients’ and doctors’ delay was found. Most important risk variables for longer patient delay were embarrassment and lower education. Most important risk variable in GP’s was ‘misdiagnosis’. TC awareness programs for men and physicians are required to decrease delay in the diagnosis of TC and improve disease free survival.

## Introduction

Current survival rates in testicular cancer (TC) are high.[1] However, delay in TC diagnosis relates to more advanced disease requiring intensive chemotherapy treatment with increased morbidity and decreased survival.[2–5] Delay in TC can be patient related or doctor related.

Until now, only a few, mainly qualitative studies, have explored delay in men diagnosed with TC.[6–8] These studies suggest that delay seems associated with men’s unawareness of the existence of TC and of warning signals such as a testicular lump or scrotal pain. Such signals may be appraised as a temporary annoyance and not serious enough to seek medical help. Also, TC affects an intimate organ in a group characterized by issues of masculinity, attractiveness, sexual functioning and other aspects of young adulthood.[9] Embarrassment to discuss testicular abnormalities could lead to delay in help-seeking behavior.[10–12] Additionally, TC mainly affects young men in a period of life when men generally do not perceive themselves as susceptible to serious disease and therefore are less likely to interpret symptoms as threatening.[12] Perceived susceptibility and perceived threat, which varies between individuals and is associated with engagement in health-related behaviors, are essential constructs in the Health Belief Model (HBM).[13]

Besides age, in cancer literature, educational level and marital status seem to be related to patient delay.[14–15] However in TC, to our knowledge only one study included education as a possible factor and reported no effect on delay and another study included marital status and found also no effect on delay.[16,17]

Delay in TC diagnosis can also be physician related, according to Andersen’s model of total patient delay.[18] In an average general practice in the Netherlands, a general practitioner (GP) will see a patient with TC once every 10 years. GPs have the important role of recognizing the relevant symptoms and providing further access to specialist care, if necessary. According to the Dutch TC guideline, patients suspected of having TC by their GP must be seen and treated by a specialist within three days.[19] In the UK exists the ‘two-week wait rules’, indicating that a patient must be seen within two weeks when urologic cancer is suspected.[20] TC diagnosis is complex because other causes than TC of scrotal swelling are more common (e.g. epididymitis, sports injuries) and patients may report complaints not associated with the testicle but caused by metastatic disease, such as fatigue, back pain and/or gynaecomastia.[10,21–23] The very low prevalence of TC, unfamiliarity with the disease, and the diversity and ambiguity of warning signals increases the chance of misdiagnosis and of delay in secondary referrals.[10,24,25]

This study aims first to gain insight into length of patients’ and doctors’ delay in TC diagnosis, and second to examine factors associated with the delay in TC diagnosis. Knowledge thus gained may provide recommendations for a timely diagnosis of TC.

## Materials and Methods

### Procedure and patients

All patients diagnosed with TC of all stages at the Department of Surgical Oncology, University Medical Center Groningen (UMCG), the Netherlands were approached to participate in this single-center, observational, quantitative study over a 3-year period. To be eligible, patients must have had sufficient command of the Dutch language. Patients with a psychiatric condition were excluded. All patients were staged with the biomarkers lactodehydrogenase (LDH), αfoetoproteine (AFP) and β-humaan choriongonadotrofine (βHCG), and with spiral computer tomography (CT) of the chest, abdomen and pelvis according to the Royal Marsden Classification and the International Germ Cell Cancer Collaborative Group (IGCCCG). Stages range from stage 1 (no evidence of metastasis) to stage 4 (evidence of extralymfatic metastasis).[26,27] Patients with Stage I disease were treated with a so called Wait and See policy. Patients with TC stages II-IV were treated with orchidectomy, cisplatin based polychemotherapy, and if indicated adjuvant surgery, eg. resection of residual disease. The surgical oncologist informed the patients diagnosed with TC on the goal of the quality of life study and provided an envelope with a questionnaire and inform consent form. Patients signed the written informed consent form, and returned the questionnaires in a prepaid return envelope. Approval of the study was granted by the UMCG Medical Ethics Review Committee (UMCG IRB 2000/027). The study was supported by a grant from The Dutch Cancer Society (RUG 99–2130).

### Instruments

A questionnaire was developed including questions on diagnostic time path and possible predictors of delay that synthesized knowledge about TC disease-specific characteristics, Andersen’s model of total patient delay, the Health Belief Model, and the interview study of Gascoigne et al.[10,13,18,28]

#### Diagnostic time path

TCPs were asked to indicate the date on which they first detected symptoms and the date of the first consultation with a general practitioner (GP) (patient delay), and the date on which they consulted a GP for the symptoms they experienced and for the first time visited a medical specialist for these symptoms(doctor delay).

#### Factors associated with delay

Patients completed questions on the following socio-demographic and illness characteristics: age, educational level, marital status, and stage of disease. Highest educational level completed was measured on a seven-point scale, ranging from primary school only (1), lower vocational degree (2), middle secondary degree (3), middle vocational degree (4), high secondary degree (5), high vocational degree (6), to university degree (7). Stage of disease (I through IV) was checked in the patient’s medical record.

Further, patients filled in questions about TC awareness (‘heard of TC’), warning signals (i.e. change in a testicle, such as a swelling or a hard lump; scrotal pain; interpretation of testicular change as cancer), limitations in daily functioning (“did you experience limitations in daily functioning because of the symptoms?”), and embarrassment about a testicular change (range 1 = not at all, 2 = a little, 3 = quite a bit, 4 = very much). Limitations in daily functioning were seen as relevant since a perceived barrier could enhance the likelihood of health-promoting behavior, such as consulting a doctor (HBM). Also, patients were asked to indicate the diagnosis made or suspected by the GP or physician they first consulted and to what disease or cause they attributed their testicular change.

### Statistical analyses

Statistical analyses were performed with SPSS 18.0 (SPSS Inc., USA). Descriptive analyses were used to calculate means, medians, frequencies and percentages. To examine factors associated with delay, Mann-Whitney U tests and Pearson’s correlations were conducted, as appropriate. Correlations with a coefficient <0.30 were considered weak, between 0.30–0.50 moderately strong, and >0.50 strong.[29]

## Results

### Participants

Sixty-one of the 66 eligible patients returned the questionnaire (response = 91%). One patient returned an almost blank questionnaire. Therefore, analyses were performed on 60 patients. Median age was 26 (range 17–45) years. Of the patients, 3.4% completed primary school only, 8.5% completed low vocational degree, 18.6% middle secondary degree, 33.9% middle vocational degree, 16.9% high secondary degree, 15.3% high vocational degree, and 3.4% had completed university. Fifty-two percent did not have a partner. Seventy-seven percent of the patients was diagnosed with extensive disease (stages II-IV) (Table 1).

**Table 1:** Descriptives and effect of patient related factors on patient delay and doctor related factors on doctor delay.

Variables (n = 60)			N (%)	Delay (days) median (range)	Correlation R/ Z-statistic	P-value
**Patient**	Median age at diagnosis (in years)		26 (17–45)		-.05 ^a^	.35
	Educational level ^c^		4 (1–7)		-.25 ^a^	.03
	Partner	Yes	29 (48)	30 (2–365)	.18 ^b^	.54
		No	31 (52)	39 (1–252)		
	Stage of disease		2 (1–4)		.33 ^a^	.01
		Stage I	14 (23)	30 (2–365)		
		Stage II	28 (47)	14 (1–120)		
		Stage III	4 (7)	136 (30–240)		
		Stage IV	14 (23)	106 (1–252)		
**Awareness**	‘Heard of TC’ before diagnosis (n = 60)	Yes	31 (52)	30 (1–240)	.02 ^b^	.98
		No	29 (48)	30 (1–365)		
**Warning signals**	Change in a testicle as a symptom (n = 60)	Yes	54 (88)	30 (1–365)	.20 ^b^	.86
		No	6 (12)	67 (1–240)		
	Pain in a testicle as a symptom(n = 54)	Yes	38 (70)	29 (1–210)	-.46 ^b^	.65
		No	16 (30)	27.5 (1–365)		
	Association of change with cancer (n = 54)	Yes	9 (17)	21 (2–56)	-1.27 ^b^	.21
		No ^d^	45 (83)	35 (1–365)		
**Limitations**	Limitations in daily living (n = 60)	Yes	25 (42)	39 (1–252)	1.13 ^b^	.26
		No	35 (58)			
**Embarrassment**	Feeling embarrassed scrotal change (n = 54)	not at all (1)	17 (38)		.79 ^a^	< .001
		somewhat (2)	19 (42)			
		quite a bit (3)	6 (13)			
		very much (4)	3 (7)			
		Missing data	9			
**Doctor**	Patients consulting with testicular change (n = 54)	TC suspected	25 (46)	1 (0–7)	5.32 ^b^	<0.001
		First otherwise diagnosed	29 (54)	14 (1–240)		
	Patients consulting without testicular change (n = 6)	TC suspected	3 (50)	2 (0–3)	1.96 ^b^	.05
		First otherwise diagnosed	3 (50)	21 (14–135)		
	Stage of disease				.28 ^a^	.04

^a^ Pearson correlation coefficient

^b^Mann-Whitney U test

^c^ highest educational level completed: primary school only (1), low vocational degree (2), middle secondary degree (3), middle vocational degree (4), high secondary degree (5), high vocational degree (6), university degree (7)

### Diagnostic time path

#### Patient delay

**Fig 1:**
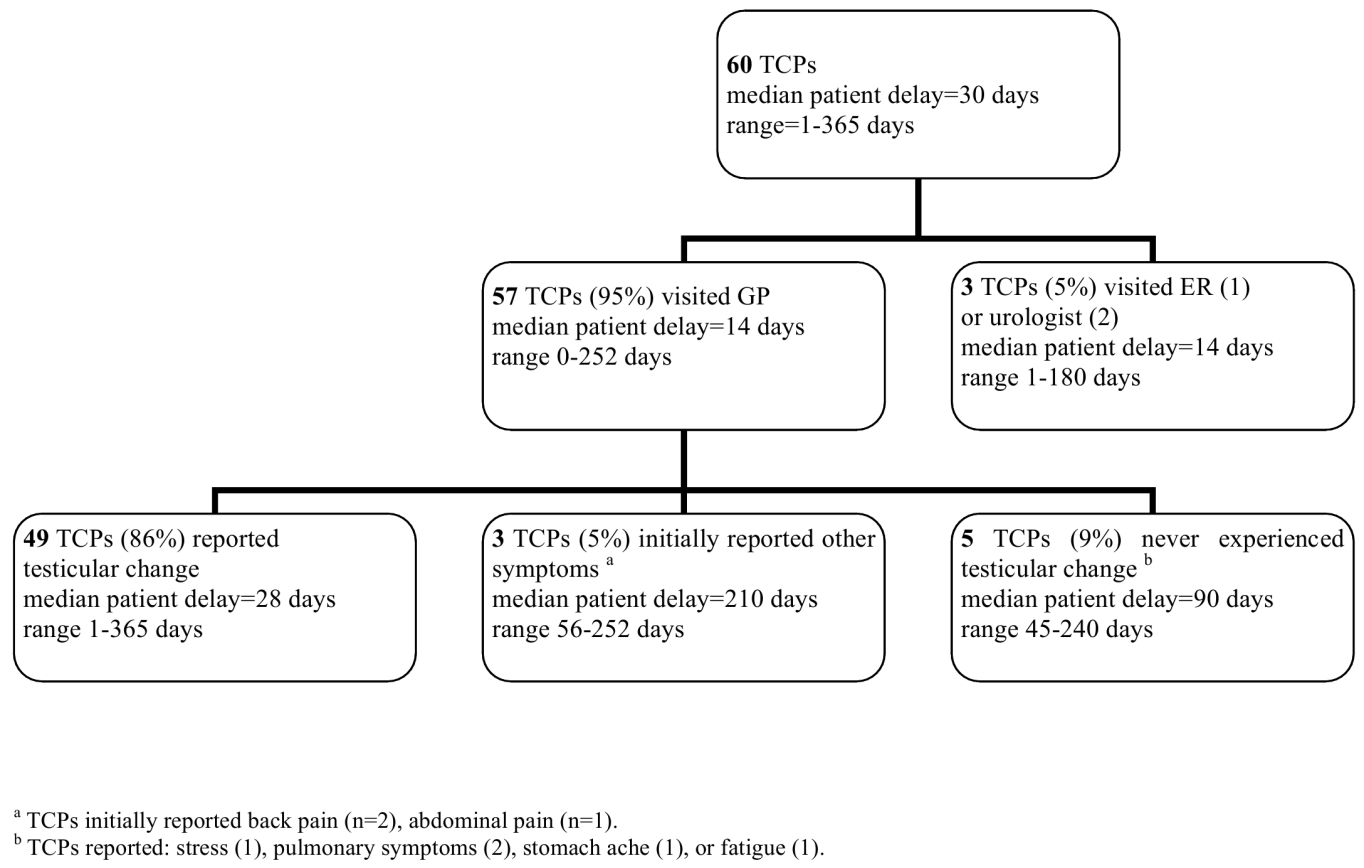
Diagnostic time path: patient delay.

(Fig 1) Median patient reported delay was 30 (range 1–365) days. Almost all TCPs (57/60, 95%) consulted the GP, the vast majority (n = 49, (86%)) for a testicular change. Three patients initially reported other symptoms, and eventually a testicular change. The remaining five patients never perceived a scrotal change. Two patients immediately consulted an urologist because of a testicular change and the last patient visited the Emergency Department with complaints other than testicular and was diagnosed with a thrombosis of the lower limb. Altogether, 6 of the 60 patients (10%) never noticed a scrotal change. Of the 54 patients noticing a testicular change, 29 patients (54%) answered that they did not consider a specific disease for the testicular change and 16 patients (30%) attributed their symptoms to diseases or causes, such as inguinal hernia (n = 3), inflammation/epididymitis (n = 5), sports injury (n = 1), Crohn’s disease (n = 2), gastritis (n = 1), hydrocele (n = 1), puberty (n = 2), or dental problem (n = 1) (9 answers missing).

#### Doctor delay

**Fig 2:**
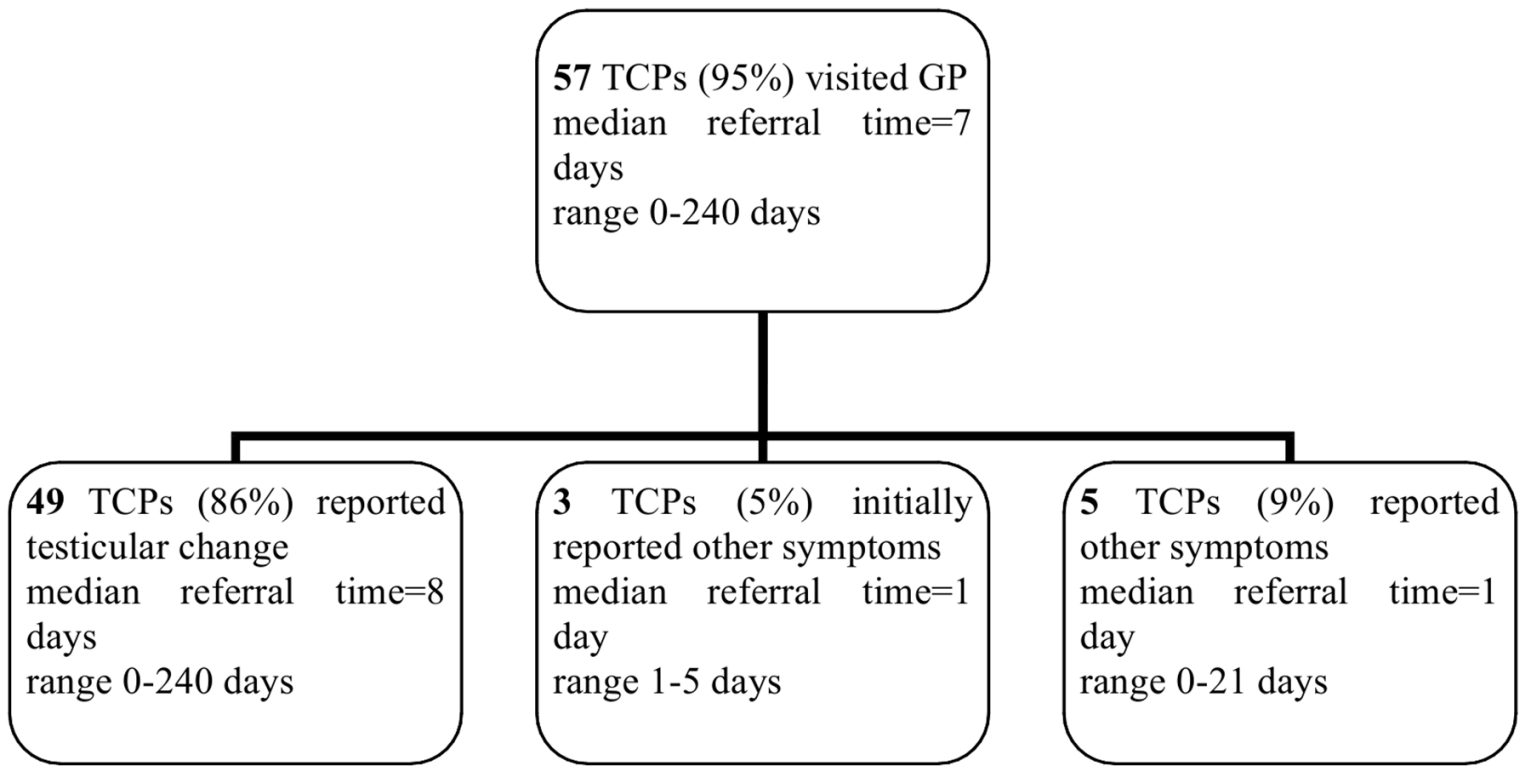
Diagnostic time path: doctor delay.

(Fig 2) Median patient remembered GPs’ referral time to a specialist was 7 (range 0–240) days. Of the 49 patients who consulted their GP because of a scrotal change at their first visit, 20 patients (41%) were referred for further examination to and seen by a specialist within 3 (median 1; range 0–3) days, 15 patients (31%) were referred and seen between 5 and 14 days, and the remaining 14 patients (28%) between 17 and 240 (median 51) days.

Of the two patients who went to a urologist, one was diagnosed with TC at day 5 and the second was first diagnosed and treated for epididymitis and diagnosed with TC at day 42. The patient who went to the Emergency Department was diagnosed with TC at day 3.

### Variables associated with patient delay

Variables possibly associated with patient delay are summarized in Table 1, and the details are summarized for patient characteristics, TC awareness, warning signals, limitations in daily functioning and embarrassment

#### Patient characteristics

No significant effect on patient delay was found of age and partnership status (r = .05 and Z = .18, respectively). Educational level was negatively but weakly correlated with patient delay (r = -.25, p = .03). A significant moderately strong positive association between disease stage and patient delay was found (r = .33, p < .01).

#### TC Awareness

Median patient delay was not significantly different between those who did (n = 31) and those who did not (n = 29) know of TC before diagnosis, nor in the total group (60 men) or in the group experiencing a testicular change (54 men). As in the total group, 48% of the men experiencing a testicular change indicated they knew of TC before diagnosis. Twelve (39%) of the 31 patients reporting they knew of TC before their diagnosis had heard of Lance Armstrong’s fight against TC.

#### Warning signals

No significant effect on patient delay was found of the experience of a scrotal change, painfulness of the testicle, and the association of scrotal change with TC. Of the 26 patients (48%) who experienced a scrotal change and who knew of the existence of TC, 17 patients (65%) did not associate their scrotal change with TC.

#### Limitations

No significant differences were found in patient delay between patients who reported limitations in daily functioning because of symptoms (n = 25) and those who experienced no limitations (n = 35).

#### Embarrassment

Of the 54 patients who reported a scrotal change, 9 did not provide an answer to the question on embarrassment. Of the remaining 45, 17 (38%) did not feel at all embarrassed about the change in their testicle, 19 (42%) felt somewhat embarrassed, 6 (14%) felt quite a bit embarrassed, and 3 patients (7%) felt very embarrassed. The relationship between embarrassment and patient delay was significant and strong (r = .79, p < .001).

### Variables associated with doctor delay

Variables possibly associated with doctor delay are summarized in Table 1, and the details are summarized for scrotal change and pain, and misdiagnosis.

#### Scrotal change and pain

The difference in patient reported doctor delay between the 54 TCPs who reported a scrotal change and the six patients who never experienced a scrotal change was not significant (Z = -.99, p = .34). Patient reported pain in a testicle had no effect on doctor referral time (Z = 1.5, p = .13).

#### Misdiagnosis

Doctor delay was significantly longer in the 29 patients (54%) who were first misdiagnosed (median = 14, range 1–240 days) than in the 25 patients (46%) immediately suspected of having TC (median 1; range 0–7 days). These 29 patients were first diagnosed by their GP with back pain (3), epididymitis (9), hydrocele (5), trauma (2), inguinal hernia (1), appendicitis (1), urinary tract infection (1), gynaecomastia (1), and in some cases no diagnoses was made (6). GP’s suspected TC in three of the six patients not reporting a scrotal change resulting in a significantly shorter referral time than in the three patients not complaining of a scrotal change and who were first otherwise diagnosed (hyperventilation, asthma, gastritis).

Finally, a positive significant weak correlation was found between doctor’s delay and disease stage (r = .28, p < .05).

## Discussion

A wide time range in patient and doctor delay was found. Patient and doctor delay were associated with more advanced disease. Delay was longer in patients who had completed a low level education and in those feeling embarrassed about a scrotal change. Longer delay in GPs was associated with initially misdiagnosing the patient.

Median patient delay in TC diagnosis in this study was 30 days, which was shorter in comparison to two studies from the eighties, but similar to a more recent study.[2,17,30] The present study displayed a wide range in patient delay. However, in contrast to the previous studies, none of the men in the present study waited longer than one year before consulting a GP.

The current study showed that educational level was significantly related to patient delay, which was in contrast to a previous study.[16] Lower educated men reported a longer patient delay. Age was not related to patient delay. It may well be that the oldest TCPs (45 years), who were still relatively young, did not feel susceptible to disease yet. Marital status was not related to patient delay, conform a previous study.[17]

Present study results show that TC awareness seems not sufficient as adequate health behaviour. Approximately half of the respondents had heard of TC before diagnosis but length of patient delay was comparable to that in patients who did not have TC awareness. In addition, neither the interpretation of a testicular change as possibly being related to cancer nor specific symptoms such as scrotal change and pain, possibly causing limitations in daily living, seemed to urge men to seek help more promptly. However, “having heard of TC” seems not to be the same as having “actual TC knowledge”.[8] Having detailed and correct information of the cause and symptoms of TC can heighten men’s disease awareness and possibly lead to an earlier GP visit.

Literature remains indistinct about the exact relevance of testicular self-examination (TSE).[31–33] The present study seem to support the view that men do not fail to detect scrotal changes but fail to act adequately upon it.[32] Nevertheless, researchers have pleaded for health education in young men to increase TC knowledge and raise awareness of the normal shape and feel of testicles, because it may encourage men to act upon scrotal changes more adequately. [2,4,34,35]

Embarrassment about the scrotal change was strongly associated with longer patient delay (p<0.001), endorsing previous suggestions that feeling embarrassed about a testicular abnormality acts as a barrier to taking action.[10,11]

Median GP referral time in patients with testicular complaints in the current study was 7 days, which was shorter than in two other studies reporting 10 and 14 days respectively, and conform a third study.[2,28,36] In the present study, two-fifth of patients with a testicular change were referred within 3 days, conform the Dutch TC guideline, and almost three-quarter were referred within two weeks, conform the current UK guideline.[19,20] Although Dutch GP’s refer a large percentage of young men reporting a scrotal change adequately, misdiagnosis seems to be a risk factor for longer doctor delay in the diagnostic process. TC is a rare disease, but GPs should always bear TC in mind, in particular when adolescents and young adult men present with inguinal or scrotal complaints, or lower back pain.

Findings of the present study accentuate the difficulty of the TC diagnostic process, and underlines the responsibility of GPs in this process. A recent English study showed that the positive predictive value for testicular cancer following a GP’s referral for a scrotal abnormality conform the two weeks rule is only 17%.[37] In the Netherlands, GPs refer patients with a scrotal mass to a radiologist for an ultrasonography. If the ultrasound is abnormal and TC is suspected, a patient is immediately referred to a surgical oncologist or urologist to confirm the diagnosis and if needed, for treatment.

The present study showed that both longer patient delay and doctor delay were significantly associated with more advanced disease, which is in concordance with other studies.[4,24] Advanced disease requires more intensive cancer treatment and is associated with increased treatment related morbidity, decreased disease free survival and increased costs.

A few limitations of the present study should be mentioned. First, methodological concerns regarding the concept of delay exist and a standardized definition is lacking. Definitions used in this study have proven operational earlier.[25] Second, patient-centered studies measuring delay are susceptible to recall bias. In particular patients reporting longer time intervals could have had difficulty remembering the exact time span. Third, to our knowledge, a validated questionnaire on this subject is not available, and thus a questionnaire incorporating information regarding TC and health behavior was developed for the present study. Fourth, the number of respondents in some subgroups is small which may affect the statistical power.

## Conclusion

High variation in patient and doctor delay was found. Patient and doctor delay were associated with more advanced disease requiring a more intensive cancer treatment. Health care providers who aim to develop education programs to increase TC awareness in young men should take into account that men who feel embarrassed about scrotal changes and lower educated men may benefit most from their programs. To prevent misdiagnoses, education programs for GPs should focus on increasing GPs knowledge of TC and on their awareness that TC may be the underlying illness in adolescent and young adult men who present with a scrotal change or with symptoms possibly considered vague. Further policy recommendations are continuous medical education of GPs to increase their understanding of the value of ultrasonography for scrotal abnormalities in combination with the biomarkers LDH, AFP and β-HCG in the differential diagnosis. Performing these diagnostics in time will increase the number of correct referrals for TC, and increase the chance that the ‘two-week wait rule’ will be met.

Both actions, education of adolescent and young adult men and of GP’s to increase knowledge and awareness of testicular cancer, and continuous medical education of GPs with respect to scrotal pathology may decrease patient’ and doctor’ delay, thus lower the percentage of TC patients diagnosed with advanced disease, decrease costs associated with treatment of advanced disease, and improve disease free and overall survival.
